# India’s Largest Hospital Insurance Program Faces Challenges In Using Claims Data To Measure Quality

**DOI:** 10.1377/hlthaff.2016.0588

**Published:** 2016-10

**Authors:** Matthew Morton, Somil Nagpal, Rajeev Sadanandan, Sebastian Bauhoff

## Abstract

The routine data generated by India’s universal coverage programs offer an important opportunity to evaluate and track the quality of health care systematically and on a large scale. We examined the potential and challenges of measuring the quality of hospital care through claims data from India’s hospital insurance program for the poor, Rashtriya Swasthya Bima Yojana (RSBY). Using data from one district in India, we illustrate how these data already provide useful insights and show that simple efforts to enhance data quality and an effort to expand the data captured could facilitate RSBY’s ability to track quality of care. The data collected by RSBY has significant potential to characterize and uncover the provision of low-quality care and help inform much-needed efforts to raise the quality of hospital care.

For more than a decade, India has expanded public health insurance coverage to over 400 million people through a variety of national and state programs. This expansion has attracted attention primarily because of the potential impacts on access, risk protection, and outcomes.^1–3^ However, these programs also generate administrative data that—in principle—can be used to evaluate some aspects of the quality of care and to design policies to tackle quality shortfalls.

Concerns about the low levels of and high variation in quality have grown alongside the expansion of coverage programs in India. Fundamental shortfalls at the primary care level range from medical professionals’ lack of formal medical training and absenteeism to low clinical quality, including incorrect diagnoses and incorrect treatment for common conditions.^4,5^

There is less systematic evidence of the quality of care at the hospital level. Doctors in public hospitals appear to exert more effort and may be less likely to prescribe antibiotics, compared to their colleagues in public clinics.^5^ Conversely, hospitalists in India spend very little time with patients, even in comparison to other developing countries.^6^ Although some specialties, such as cardiac care, appear to have high rates of appropriate use in specific contexts,^7^ there appears to be substantial variation in quality more generally.^6^

The use of claims data offers an important opportunity to evaluate quality systematically and on a large scale. Countries with established insurance systems have long recognized the potential of such data to monitor performance and quality and to inform health policies. For example, in the United States and most Organization for Economic Cooperation and Development countries, public and private payers make extensive use of routine data.^8^ In addition, organizations in the United States such as the Health and Medicine Division of the National Academies of Sciences, Engineering, and Medicine (previously called the Institute of Medicine) conduct rigorous research on quality gaps, and organizations such as the National Quality Forum develop and curate standards and measures.^9^

In contrast, data systems in middle-income countries are much less developed, and efforts to systematically measure and improve quality remain in their infancy. Some public health insurance programs in India generate data that can be used to examine such topics as the appropriate use of specific procedures^7^ and the occurrence of infections and hospital readmissions.^10^ However, in middle-income settings, claims are primarily used to serve the narrow operational needs arising from processing payments.

We examined the potential for and challenges to measuring quality using claims data from the Rashtriya Swasthya Bima Yojana (RSBY, National Health Insurance Plan), a program for the poor in India. RSBY is India’s largest government-sponsored health insurance program and currently covers about forty million families.^2^ We reviewed the structure and quality of RSBY claims data and used data from a district in the state of Orissa for the period September 2013–January 2014 to illustrate how these data might be used to study hospital quality. We use the resulting insights to discuss opportunities to modify the RSBY data system to enable closer monitoring of quality and inform policy design.

## Background

RSBY was started in 2008 in the context of the challenges faced by the public sector in meeting demand for health care services and improved performance in providing care, and the concurrent rise of the private sector as a major provider of inpatient and outpatient services. For example, in 2014 the private sector provided 72 percent of treatments in rural areas and 79 percent in urban areas.^11^ RSBY was designed to address concerns that lower-income families were at risk of substantial out-of-pocket spending, especially for hospitalization in the private fee-for-service system.

RSBY covers all families with incomes below the Indian poverty level and some categories of unorganized workers who generally lack access to formal social protection such as health insurance and pensions. Beneficiaries of RSBY can choose from among a network of hospitals in the public and private sectors that are contracted (empaneled) to provide covered services without the beneficiary having to pay any cost sharing. The program is “cashless” at the point of service: Providers are reimbursed according to a regulated fee schedule by competitively selected insurance companies that operate the program for the government.

RSBY introduced various innovations in management and operations in India—most importantly, moving to an electronic platform for enrolling beneficiaries, registering patients at hospitals, and settling claims. Data are captured at different stages, including at enrollment in the program and at each admission and discharge, and linked through unique record numbers in a centralized database. Each family receives a “smart card” that records family members’ fingerprints and is used to authorize treatment in a hospital at admission and to process the final claim at discharge. At admission, a claim receives a unique identification number and is submitted electronically by the hospital for reimbursement; the claim is settled by the insurer after the patient has been discharged. The insurer submits a core set of transactions data to the state and national governments for financial approval and reimbursement.

The current focus of RSBY is on frequent secondary-level inpatient procedures, such as cataract surgery and gall bladder removal. The program covers hospitalization expenses for medical and surgical procedures up to a cap of 30,000 rupees (about US$450) per year for a family. RSBY does not use preauthorization but checks at admission whether the cap has been reached. Benefits for hospitalizations are available to patients who are admitted to an empaneled hospital for at least twenty-four hours or who undergo specified surgeries or procedures that do not require this minimum hospital stay.

The most commonly performed procedures have a predetermined “package rate,” a case-based payment that is the same for all hospitals in a specific geographical region (a “package” is a discrete hospital-based treatment or procedure with an initiation and termination date). Non-surgical diagnoses are reimbursed through a daily rate. Recently, some Indian states have added additional or bonus payments for hospitals that have secured accreditation for complying with defined quality standards. At the national level, the recent shift of RSBY to the Ministry of Health and Family Welfare allows for a closer coordination between it and the ministry’s flagship program, the National Health Mission.

## Study Data And Methods

**Data** We used transactions data based on claims for which the contracted insurance company paid empaneled hospitals for RSBY services to beneficiaries residing in Puri District, in the eastern state of Orissa, for the period September 2013–January 2014. The data were provided by the Ministry of Labour and Employment, as part of our study of a pilot program to expand outpatient care through RSBY. The data contain each patient’s age and sex, dates of and status at admission and discharge, whether the patient died in the hospital, final diagnosis, procedure categories, package codes and names, and final payment amounts (the full list of data fields is available in the online Appendix).^12^ We matched the package codes to the RSBY fee schedule that was used in Orissa in the period July 2011–August 2014 to obtain the standard fee and expected length-of-stay.

We focused on hospitals with 40 or more claims from September 2013 to January 2014. Thus, our analytical data included 3,437 unique claims from 20 hospitals, out of the full data set of 3,712 claims from 51 hospitals.We grouped 11 procedure categories with fewer than 40 claims each (a total of 134 claims) into an “other” category to facilitate exposition.

We assessed the structure and quality of the RSBYdata through comparisons with Medicare’s form CMS-1450, which US hospitals can use to submit claims in the fee-for-service program.^13^ We examined the quality of existing RSBY data fields by assessing the completeness and legibility of free-text fields.

**Examining Variation** We examined variations in length-of-stay in vaginal hysterectomy—the package that was the largest in terms of the number of claims, excluding the “general ward”package, which is used for a wide variety of procedures. We calculated length-of-stay as the difference between admission and discharge dates.

The absence of an accepted quality framework in India remains a key constraint. With no national benchmarks for what is an appropriate length-of-stay for a given procedure, we had to benchmark our analysis based on RSBY’s data. We therefore calculated the share of claims that exceeded the length listed in the RSBY fee schedule. We omitted the 6 percent of claims that had lengths-of-stay that were three or more times longer than the median for their package code, since these lengths-of-stay could be the result of connectivity issues or data entry errors.

We also examined variation in the procedures and patients (by age and sex) across hospitals, as well as in total RSBY payments. We categorized age into two groups—younger than age forty and age forty and older.We calculated the procedure-specific median claim value and length-of-stay because the underlying distributions were skewed.

**Limitations** Our study had important strengths and limitations. The strengths include our use of unique administrative data that captured all hospital utilization reimbursed through RSBY for beneficiaries residing in Puri. Fields critical to financial approvals were checked by the RSBYinsurers to confirm that they were complete and correct and to mitigate fraud and associated liabilities. Also, our assessment of the data structure is generalizable to other states participating in RSBY, since RSBY uses the same data collection system, including data fields and data collection software, in all Indian states.

The potential of linking with existing RSBY data could also motivate the development of new systems.

The study’s major limitations were its limited time period and the fact that the sample might not be representative of the empaneled hospitals’ patients or of utilization by RSBY beneficiaries in other areas in Orissa or India. In particular, because our data originated from a single district, the empirical findings from our analyses are not generalizable beyond our study setting. We therefore used these analyses to illustrate the potential and challenges of using RSBY claims data to measure quality, and to complement our assessment of the data system.

In addition to these major limitations, several data fields might not have been externally verified, and the data in them could be unreliable.We had no way to establish possible error rates, but we note that insurers are asked to monitor claims and conduct on-site spot checks, and they depend on these data to process payments.

Also, current RSBYclaims data do not allow for a detailed study of symptoms, diagnoses, and treatments, which precluded more detailed analyses of hospital quality. This was not unexpected, given that the primary function of these data is to support financial approvals of claims. Consequently, while data on lengths-of-stay provide some indication of possible quality problems, on their own the data lacked adequate depth and breadth to enable us to make credible assessments of potential issues—including inefficiencies and underuse, overuse, or misuse of care—that have been identified in settings such as the United States.^9^

## Study Results

**Comparing Claims Data** Overall, the RSBY data contain fewer fields and fewer details, compared to data from Medicare’s form CMS-1450.^13^ For example, the final RSBY diagnosis field is a single free-text field, whereas Medicare collects multiple primary and secondary diagnoses using standard codes. Similarly, the RSBYdata contain no information on many of elements that are present in the Medicare form, including admission type (for example, elective or emergency); the identity of the attending provider; and which procedures, tests, and prescriptions the patient received. In general, Medicare has a high level of specificity for most of its data fields, such as requiring up to seven digits for the principal procedure code.

In RSBY claims data, fields that are mandatory and system-generated, such as dates and package codes, were always filled in. We could not assess whether these fields were correctly filled in—for example, whether providers used the correct package code to describe their clinical activities. The mortality field was almost always filled in (it was not filled in for only four claims, or 0.1 percent of the claims in our study), but death in the hospital was reported for 0.5 percent of claims, all of which were associated with the “general ward” package. The unstructured text fields for patient status at admission and discharge, final diagnosis, and mortality summary were not usable as recorded in the raw claims. These entries were not systematically structured and were often uninformative. For instance, the most frequent entry in the mortality summary was “patient is dead.”

**Characteristics Of Claims From Puri** Fifty-seven percent of the claims in our sample were categorized as “medical” (also referred to as “general ward”) procedures, and there were also large percentages of claims for gynecology (17 percent) and general surgery (16 percent) (calculated from data in Exhibit 1). These three categories also received most of the payments in the RSBY data—collectively, about 79 percent of the 17.8 million rupees paid out.

**EXHIBIT 1:** Claims data from RSBY for Puri District, Orissa, India, September 2013–January 2014

	No. of claims	No. of packages in claims	No. of packages in schedule	Median LOS (days)	Median payment (rupees)	Total payments (rupees)
**Claims by procedure category**					
Medical	1,951	2	2	4	2,000	4,696,375
Gynecology	593	24	53	5	10,000	4,707,031
General surgery	565	63	344	3	8,750	4,591,811
Combined packages	81	10	28	5	15,000	1,181,000
Endoscopic procedures	63	9	29	3	11,000	786,300
Urology	50	25	119	4	12,000	538,750
Other	134	48	462	4	7,500	1,290,375
All categories	3,437	181	1,037	—^a^	—^a^	17,791,642
**Claims by package (top five packages by number of claims)**					
General ward, unspecified	1,935	—^a^	—^a^	4	2,000	4,612,375
Vaginal hysterectomy	316	—^a^	—^a^	5	10,000	3,157,500
Normal delivery	131	—^a^	—^a^	2	2,500	327,500
Fissurectomy and hemorrhoidectomy	82	—^a^	—^a^	3	11,250	919,687
Hernia repair and release of obstruction	54	—^a^	—^a^	3	10,000	540,000

**SOURCE** Authors’ analysis of claims data from Rashtriya Swasthya Bima Yojana (RSBY) for Puri District, Orissa, India.

**NOTES** Packages are explained in the text. The fee schedule for RSBY includes 1,090 codes, but 53 codes in the medical category are not associated with payments—instead, they are paid through a general ward daily rate (500 rupees). Total payments are the sum of payments for a procedure category or package. LOS is length-of-stay.

a Not applicable.

The fee schedule listed only two packages in the medical procedure category that were associated with per day payments—“general ward, unspecified” and “intensive care unit.” The schedule included fifty-three packages in gynecology, of which twenty-four were represented in our data. Across procedure categories the top package was “general ward, unspecified,” which accounted for 56 percent of claims. The next two largest packages were in the gynecology category—vaginal hysterectomy (9 percent) and normal delivery (4 percent) (calculated from data in Exhibit 1).

**Variations** There was substantial variation in lengths-of-stay for vaginal hysterectomy. The length-of-stay indicated in the RSBY fee schedule appeared most frequently in the data. However, there was a long right tail in the distribution of length-of-stay (Exhibit 2), meaning that more than half (52 percent) of hysterectomy claims exceeded the fee schedule’s length-of-stay (data not shown).

**EXHIBIT 2:**
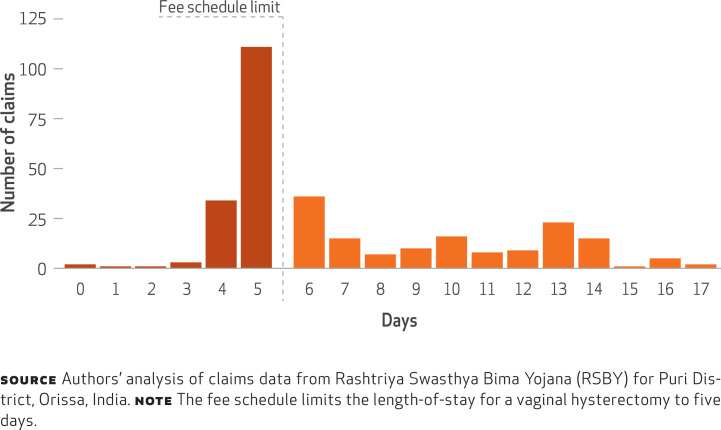
Lengths-of-stay for vaginal hysterectomy relative to length-of-stay in the RSBY fee schedule, for Puri District, Orissa, India, September 2013–January 2014

**SOURCE** Authors’ analysis of claims data from Rashtriya Swasthya Bima Yojana (RSBY) for Puri District, Orissa, India. **NOTE** The fee schedule limits the length-of-stay for a vaginal hysterectomy to five days.

Similarly, there was substantial variation in the volume of claims and patient mix across hospitals (Exhibit 3). The top three hospitals in terms of number of claims accounted for almost half of all claims but only one-third of the total RSBY reimbursement. In addition, hospitals that submitted claims for medical or “general ward”procedures tended to have a large share of claims falling in that category. We lacked the further data—for example, information that would allow us to distinguish between specialty and general hospitals—required to examine these patterns.

**EXHIBIT 3:** Claims data and patient mix, ranked among the top 20 hospitals in Puri District, Orissa, India, September 2013–January 2014

Rank	No. of all Claims	Medical claims (% of total)	Patient characteristics		Median LOS (days)	Median payment (rupees)	Total payments (rupees)
			Male	Ages 40 and older			
1	796	97	62%	67%	3	1,500	1,611,475
2	671	66	37	77	7	4,000	3,814,225
3	197	82	45	75	4	2,500	697,700
4	191	66	61	71	4	3,000	915,906
5	152	0	45	75	4	10,000	1,369,925
6	145	4	37	79	4	10,000	1,331,800
7	142	6	32	40	3	10,000	1,379,187
8	134	100	72	66	4	2,000	268,000
9	130	23	37	75	4	10,000	1,075,000
10	120	10	63	70	3	7,000	764,187
11	97	3	22	77	5	10,000	980,750
12	94	0	2	0	2	2,500	235,000
13	88	92	48	67	2	1,000	103,500
14	86	0	31	74	4	11,250	1,009,175
15	84	0	54	87	4	10,000	774,750
16	81	85	64	65	5	2,500	316,000
17	71	92	38	51	3	1,500	98,750
18	63	65	35	41	2	1,000	89,500
19	48	0	6	54	4	10,000	460,062
20	47	4	55	68	4	11,250	496,750

**SOURCE** Authors’ analysis of claims data from Rashtriya Swasthya Bima Yojana (RSBY) for Puri District, Orissa, India.

**NOTES** Total payments are the sum of payments to a hospital. LOS is length-of-stay.

## Discussion

**Using Data To Measure Quality** Our assessment of the RSBY data structure indicates many opportunities to use these data to examine quality and inform program design. Although less comprehensive than Medicare’s claims data, the RSBY system data, in principle, capture key information, including patient data (that can be linked to enrollment files) and information on procedures, diagnoses, and even mortality. There are legitimate operational reasons for collecting fewer details than Medicare’s fee-for-service program does, including the fact that RSBY pays by package (not by service) and its limited capabilities for collecting data—as well as the costs of collecting them.

However, the way in which data are captured in practice renders many of the existing fields unusable, as our empirical extract of data from Puri demonstrates. In particular, entries in free-text fields are not structured, nor do they contain specific information that could be extracted and categorized after data entry. As result, although data elements exist to support first-order quality measures such as risk-adjusted mortality rates, in practice these measures cannot be reliably derived from the existing data. An easy first step toward solving this problem would be to implement prepopulated lists of categories, mandatory fields, and software checks—for example, flags for likely mismatches between diagnosis and procedure codes.^7^

Even our basic analyses suggested that there were substantial variations in the care patients received.

However, improving the quality of data entry may also require substantial training of entry clerks at hospitals. Data quality problems persist in highly developed systems, including those in the United States, despite decades of investment.^14^ RSBY can learn from these experiences to leapfrog this evolution, just as it has done with regard to the use of smart cards and identification systems for beneficiaries.

The claims data comparison between RSBY and Medicare also indicates valuable opportunities for expanding the contents of RSBY’s claims form. RSBY should consider increasing the level of detail in its coding by allowing the use of multiple principal diagnoses; recording additional details on procedures (beyond the package code); and capturing patients’ types of admission (for example, elective or emergency), treatments, and prescription drugs. This information would facilitate routine analyses of appropriate care and help track overuse and underuse of care, as well as treatment patterns. Some analyses, such as examining readmissions, would also be in the interest of the insurance companies. There is precedent for such claims analysis in India.^7^ RSBY could slowly move in this direction—for instance, by progressively increasing the coding detail.

Finally, RSBY could improve its use of the smart cards and patient identifiers to link beneficiaries across programs and databases with the goal of overcoming problems with RSBY’s internal data sources or obtaining additional details. For example, linkages to RSBY enrollment files could provide socioeconomic information on patients, such as their level of education, occupation, and designation as a member of a scheduled tribe or caste. Current RSBY enrollment processes collect limited information, such as members’ age and sex. Further information could be included at the enrollment stage to allow for more nuanced analyses of claims and experiences of health care quality by demographic groups without requiring providers to collect more detailed information at the point of service.

Similarly, tracking beneficiaries across other government programs and databases could provide insights on the design of RSBY as well as other important public policy issues, such as whether participation in assistance programs mitigates the economic impact of health shocks.

Taking advantage of these opportunities would require coordinated action at a level higher than RSBY’s management to create procedures that would facilitate data linkages and to alleviate bottlenecks and data problems that are likely to exist in other programs. The priority should be on linking claims data with data currently available to RSBY (such as data in enrollment files) or relatively easy to obtain (for example, data already collected by insurers but not now passed on to RSBY, or data from state health insurance programs—such as the one in Meghalaya—that use the RSBY platform). More substantial efforts will be required to link RSBY’s data to other programs that may use their own identification systems and databases. The potential of linking with existing RSBY data could also motivate the development of new systems, such as a comprehensive death record system.^15^

**Substantial Variation Across Packages And Hospitals** Our case study of lengths-of-stay across procedures and of procedure and patient mix across hospitals illustrates the value that routine claims analyses could offer to RSBY. The overall patterns suggest a lack of specificity in coding, since more than half of all claims were for “general ward, unspecified.” The relatively high volumes of vaginal hysterectomies could also be concerning, especially if they were performed in a short period of time. Unfortunately, we lack the additional details needed to examine this question.

The large variation in lengths-of-stay and the large share of stays that exceeded the RSBY standard could indicate inefficiencies or reflect heterogeneity in stays. In the latter case, RSBY might need to consider varying reimbursements according to case-mix.

Similarly, the hospital profiles we found could have implications for the design and management of the RSBY provider network. In Puri a few hospitals accounted for most of the claims and received most of the reimbursement money. This could be a result of a limited network that channeled patients to a few hospitals. RSBY could examine reasons for this variation and investigate whether it has implications for quality and whether a larger network is required to provide adequate access—and, if so, how one could be developed. A related question is whether specific quality measures vary across hospitals with small and large volumes of care. For example, low volumes of specialty care may be associated with worse outcomes.^16^

However, these are questions that need further study. Our RSBY data did not capture the hospitals’ full volume of claims, and we did not observe hospital characteristics, such as whether a hospital provided only selected specialty care.

Overall, even our basic analyses suggested that there were substantial variations in the care patients received. This, in turn, suggests moving from asking whether variations exist to asking why they exist, which is not a negligible step forward for programs such as RSBY. A logical next step would be to expand the analysis to a larger context, such as an entire state, and to examine differences in case-mix, efficiency, and other factors.

## Conclusion

The absence of an accepted quality framework and national benchmarks in India remains a key constraint to the systematic measurement of quality, starting with data capture at the provider level. RSBY and, more recently, the National Health Mission (through the National Health System Resource Centre) are working to develop standard clinical pathways for commonly performed procedures.^17^ However, implementation of these pathways will take time and will be challenging. In the meantime, existing data sources such as claims data may be the best tool to use for routine quality measurement.

In this spirit, we have reported here on the current capabilities and limitations of using claims data from India’s largest insurance program to monitor and study the quality of hospital care, as well as opportunities for improving and extending the data that are captured. RSBY’s current claims data fall short in several areas, but many of the problems could be easily addressed.

Obvious areas for immediate improvement include replacing free-text fields with structured fields and imposing automated logical checks. Improving the training of data entry clerks could also improve the quality of the existing data. Future updates to the data system could introduce additional data fields, including those related to diagnoses, procedures, and medications. Because capturing the additional information would impose costs on hospitals, RSBY could consider providing incentives to hospitals to meet benchmarks in reporting. Finally, the individual identifiers embedded in beneficiaries’ smart cards could enrich RSBY data through links to records from other programs, at least on the state level and once data systems have been harmonized.

The absence of an accepted quality framework and national benchmarks in India remains a key constraint to the systematic measurement of quality.

There is great potential for simple improvements and learning from the best practices of advanced systems, including Medicare. RSBY has proven adept at leapfrogging many highly developed systems in related areas, such as the use of electronic enrollment records and smart cards. There is similar potential for improving the program’s claims data, as well as for using routine data for new initiatives. For example, RSBYcould use mobile phone numbers captured during the enrollment and transaction processes to implement dynamic feedback systems, such as conducting rapid verification of services provided to beneficiaries and gauging patient satisfaction. In the medium term, the program could train participating clinicians on clinical pathways, insist that they be followed, and monitor how hospitals perform vis-à-vis the pathways.

In addition, and despite many problems with the data, RSBY’s current claims data can be used to track basic quality metrics such as length-of-stay, as we did in this study, and can be used to create basic hospital profiles. Although properly explaining variations in these measures requires more data of better quality than we had available, our findings suggest that even the limited existing data contain valuable information. RSBY, as well as state and federal governments, should make maximum use of this readily available resource, continue to improve the capture of data, and build in high-value additions. Doing so would facilitate systematic and routine tracking of the quality of care at all empaneled hospitals, and it would create an important evidence base with which to tackle India’s many challenges in providing high-quality health care services.

This study builds on an earlier collaboration, using the same data, between the World Bank and the Government of India. The associated analytical work was cofunded by the UK Department for International Development and the World Bank. The views expressed in the article do not necessarily reflect the official policies of the UK Government or the World Bank Group. Rajeev Sadanandan was previously the joint secretary and director general for labor welfare in the Indian Ministry of Labour and Employment, where he was in charge of the Rashtriya Swasthya Bima Yojana program.

## NOTES

1 Sood N, Bendavid E, Mukherji A, Wagner Z, Nagpal S, Mullen P. Government health insurance for people below poverty line in India: quasi-experimental evaluation of insurance and health outcomes. BMJ. 2014;349:g5114.

2 La Forgia G, Nagpal S. Government-sponsored health insurance in India: are you covered? [Internet]. Washington (DC): World Bank; c 2012 [cited 2016 Aug 12]. Available from: http://documents.worldbank.org/curated/en/644241468042840697/pdf/722380PUB0EPI008029020120Box367926B.pdf

3 Karan A, Yip W, Mahal A. Extending health insurance to the poor in India: an impact evaluation of Rashtriya Swasthya Bima Yojana on financial risk protection [Internet]. Rochester (NY): Social Science Research Network; 2015 Dec 11 [cited 2016 Aug 12]. Report No. ID 2702395. Available for download from: http://papers.ssrn.com/abstract=2702395

4 Das J, Holla A, Das V, Mohanan M, Tabak D, Chan B. In urban and rural India, a standardized patient study showed low levels of provider training and huge quality gaps. Health Aff (Millwood). 2012;31(12):2774–84.

5 Das J, Hammer J. Money for nothing: the dire straits of medical practice in Delhi, India. J Dev Econ. 2007;83(1):1–36.

6 Das J, Hammer J, Leonard K. The quality of medical advice in low-income countries. J Econ Perspect. 2008;22(2):93–114.

7 Sood N, Ugargol AP, Barnes K, Mahajan A. Applying appropriate-use criteria to cardiac revascularisation in India. BMJ Open. 2016;6(3): e010345.

8 Organization for Economic Cooperation and Development. Strengthening health information infrastructure for health care quality governance: good practices, new opportunities, and data privacy protection challenges. Paris: OECD; 2013 May 15.

9 Institute of Medicine. Crossing the quality chasm: a new health system for the 21st century. Washington (DC): National Academies Press; 2001.

10 Sood N, Wagner Z. Impact of health insurance for tertiary care on post-operative outcomes and seeking care for symptoms: quasi-experimental evidence from Karnataka, India. BMJ Open. 2016;6(1):e010512.

11 Indian National Sample Survey Office. Key indicators of social consumption in India: health [Internet]. New Delhi: Ministry of Statistics and Programme Implementation; 2015 [cited 2016 Aug 15]. Available from: http://mospi.nic.in/mospi_new/upload/nss_71st_ki_health_30june15.pdf

12 To access the Appendix, click on the Appendix link in the box to the right of the article online.

13 Centers for Medicare and Medicaid Services. Medicare claims processing manual: Chapter 25—completing and processing the Form CMS-1450 data set [Internet]. Baltimore (MD): CMS; [revised 2015 Dec 31; cited 2016 Aug 15]. Available from: https://www.cms.gov/Regulations-and-Guidance/Guidance/Manuals/downloads/clm104c25.pdf

14 Peabody JW, Luck J, Jain S, Bertenthal D, Glassman P. Assessing the accuracy of administrative data in health information systems. Med Care. 2004;42(11):1066–72.

15 Jha P, Gajalakshmi V, Gupta PC, Kumar R, Mony P, Dhingra N, et al. Prospective study of one million deaths in India: rationale, design, and validation results. PLoS Med. 2006;3(2):e18.

16 O’Sullivan M. Safety in numbers: cancer surgeries in California hospitals [Internet]. Oakland (CA): California Health Care Foundation; c 2016 [cited 2016 Aug 15]. Available from: http://www.chcf.org/publications/2015/11/safety-cancer-surgeries-hospitals

17 National Institute for Health and Care Excellence. Towards national standard treatment guidelines (STGs) for India [Internet]. London: NICE; c 2016 [cited 2016 Aug 24]. Available from: https://www.nice.org.uk/about/what-we-do/nice-international/nice-international-projects/towards-national-standard-treatment-guidelines-stgs-for-india

